# Comparative analysis of clinical characteristics of symptomatic pituitary adenomas in elderly patients: a consecutive series of 114 patients

**DOI:** 10.3389/fendo.2024.1385813

**Published:** 2024-12-06

**Authors:** Run Wang, Xiaodi Han, Cheng Xie, Qinghua Zhang, Liang Kan, Sheng Han

**Affiliations:** ^1^ Department of Neurosurgery, Huazhong University of Science and Technology Union Shenzhen Hospital (Nanshan Hospital), Shenzhen, China; ^2^ Department of Neurosurgery, The First Hospital of China Medical University, Shenyang, China; ^3^ Department of Geriatrics, Shengjing Hospital of China Medical University, Shenyang, China

**Keywords:** pituitary adenomas, elderly patients, visual impairment, tumor apoplexy, retrospective study

## Abstract

**Background:**

Pituitary adenomas (PAs) present with clinical features such as neuroendocrine abnormalities and mass effects, common in the general morbidity population. However, in elderly patients, the disease progression renders some clinical features difficult to detect and identify in time. Consequently, elderly patients with PAs are often not identified and receive sufficient intervention on time to achieve a satisfactory outcome.

**Methods:**

Clinical data were collected from 114 consecutive patients older than 70 years with PAs who had undergone surgery. Based on the average age, the patients were categorized into a younger group and an elder group, and were statistically analyzed and compared.

**Results:**

Sixty-five males (57.0%) and 49 females (43.0%) were included in the study, with an average age of 73.2 years. Their common preoperative symptoms included vision impairment, followed by headache and vomiting, and visual field defect. The milder the preoperative visual impairment, the greater the possibility of post-operative visual improvement (P=0.001). The tumors were primarily non-functional pituitary adenomas (NFPAs, 73.7%), with a high degree of suprasellar invasion but a low degree of parasellar invasion (P<0.0001). For further analysis, based on the average age, we categorized the patients into younger (age< 73 years, 59 cases) and elder (age≥ 73 years, 55 cases) groups. The elder group was more likely to have preoperative vision impairment problems (P=0.044), whilst post-operative visual improvement was worse (P=0.001). The elder group also had a more severe suprasellar invasion (P=0.009), with a higher proportion of NFPA than the younger group (P=0.006). Compared to the younger group, the tumors in the elder group were larger (P=0.039), and had a higher rate of apoplexy (P=0.039), and were more likely to have comorbid postoperative complications (P=0.031), such as fever and cerebrospinal fluid (CSF) leakage, compared to the younger group.

**Conclusions:**

Elderly patients with PA had specific clinical characteristics. Their relatively worse pre- and post-operative conditions and intraoperative findings illustrated the need for early surgery.

## Introduction

Pituitary adenomas (PAs) are benign tumors originating from the anterior pituitary gland, with an incidence of 3.9 to 7.4 cases per 100,000 (1). PAs are typically detected as they cause abnormal hormone secretion or mass effect, producing clinical symptoms (2). The clinical features of PAs vary across different age groups. PAs have been found to be more likely to cause hormone-related symptoms in younger patients (3), while they are often mass effect or hypopituitarism in elderly patients (4, 5). PAs in the elderly account for 10-15% of all cases, with increasing detection rates as people aspire for a better quality of life (5–7). The detection of the mass effect or hypopituitarism in elderly patients with PA is often delayed, thus affecting prognosis (5, 7). In the elderly with PAs, transsphenoidal surgery (TSS) is generally considered to be the preferred and safe option; nonetheless, major controversies still exist regarding the improvement of preoperative symptoms (in terms of visual acuity) and the recovery of hypopituitarism and tumor recurrence (8–13). Therefore, this single-center retrospective study aimed to analyze the unique clinical characteristics of PA in elderly to facilitate better identification and timely intervention in such patients.

## Methods

### Patients

A total of 114 consecutive elderly patients (over 70 years old) diagnosed with PA underwent surgery in the Department of Neurosurgery, The First Hospital of China Medical University, between January 2011 and February 2019. All patients underwent transnasal endoscopic or microscopic surgery. Approval for this study was obtained from the institutional review board at The First Hospital of China Medical University. Written informed consent was obtained from each patient or their relatives for using their clinical data for a retrospective study.

### Clinical examinations

A detailed medical history, including the history of previous illnesses, smoking, and alcohol consumption, of all patients was taken. On admission, pre-operative investigations were routinely performed, including hormone tests to determine the type of PA and magnetic resonance imaging (MRI) of the sellar region to determine tumor morphology. Visual disability included vision impairment as measured by an eye chart and visual field defect as measured by computerized perimetry. The visual impairment score (VIS) was used to evaluate visual disability caused by compression of the optic nerve and chiasma (14). The surgical risk was categorized by American Society of Anesthesiologists (ASA) score, scaled from I to VI, according to physical status and overall health of the patients. The degree of tumor invasion to the supra- and para-sellar region, were graded, respectively, through Hardy-Wilson classification and Knosp classification (15, 16). Further, tumor volume was approximated using the ellipsoid formula (V=πabc/6, a, b, c were anteroposterior diameter, transverse diameter, and axial diameter, respectively). Intraoperative information on clinical features, post-operative review of hormone levels, and post-operative complication data were also collected and analyzed. The intergroup comparisons were also made.

Near-total resection (NTR) means the removal of at least 90% of the tumor, whereas subtotal resection (STR) means that less than 90% of the tumor has been removed. Abnormal hormone levels were assessed based on baseline hormone levels with the following normal range: luteinizing hormone (LH): 6-34 mIU/mL; follicle-stimulating hormone (FSH): 2-22 mIU/mL; prolactin (PRL): 1.5-30 μg/L; growth hormone (GH): 0.05-8 μg/L; insulin-like growth factor-I (IGF-1): 10-500 ng/mL; adrenocorticotropic hormone (ACTH): 7.2-63.3 pg/mL; cortisol (COR): 171-536 nmol/L forenoon, and 64-327 nmol/L afternoon.

Tumor classification follows the World Health Organization 2017 classification (17) of tumors of the pituitary, and is combined with clinical symptoms and pre-operative hormone tests (18). Patients with preoperative PRL levels greater than 250 μg/L were diagnosed with lactotroph adenomas. Patients with elevated IGF-1 levels (>750 ng/mL) detected twice preoperatively, and lack of suppression of GH levels during an oral glucose tolerance test, were diagnosed with somatotroph adenomas. Patients, after underwent cortisol and dexamethasone suppression test to prove hypercortisolism, were diagnosed with corticotroph adenomas when sellar mass was detected as well as ectopic corticotropin secretion was excluded. Patients with one or more pituitary hormones deficiency measured by postoperative serum hormone level were diagnosed with hypopituitarism (19). Post-operative pathology and immunohistochemical analyses were conducted by the Department of Pathology, The First Hospital of China Medical University.

### Statistical analysis

The chi-square test and t-test were used to identify statistical differences in the data of interest and to compare the two groups. Statistical analysis was performed using SPSS v25.0 (SPSS Inc., Chicago, IL). Differences were considered statistically significant at P<0.05.

## Results

### Clinical features

**Figure 1:**
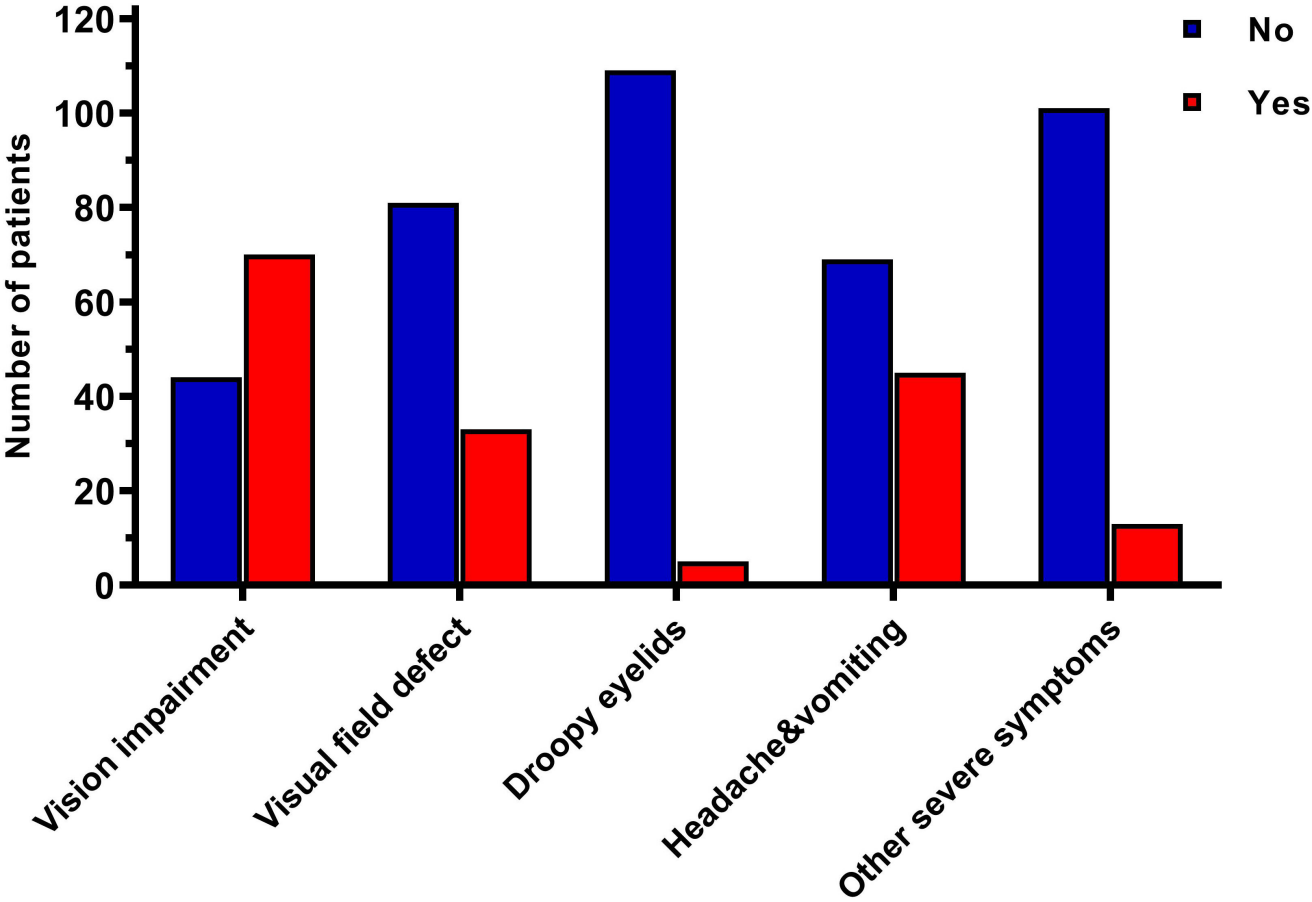
Differences in clinical symptoms of 114 elderly patients.

**Figure 2:**
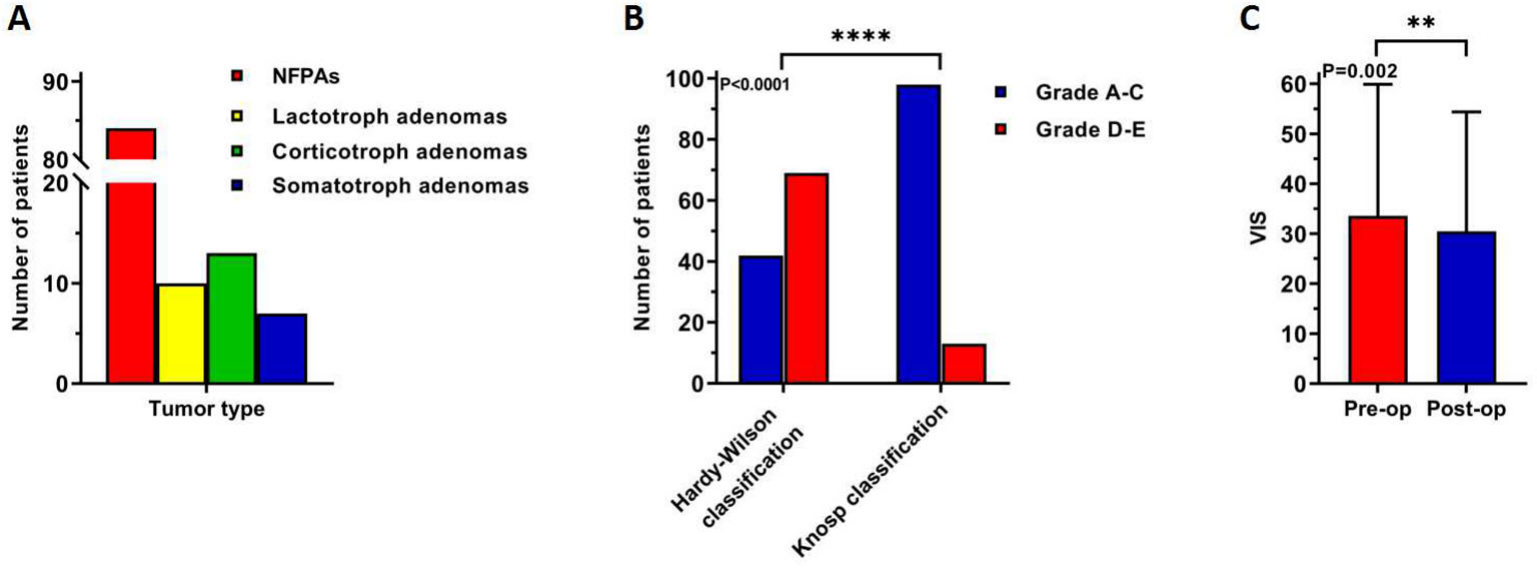
Difference in clinical characteristics of 114 elderly patients: **(A)** tumor type, **(B)** a high degree of suprasellar invasion while a low degree of parasellar invasion, **(C)** post-operative visual impairment was improved. **: P<0.01; ****: P<0.0001.

A total of 65 males (57.0%) and 49 females (43.0%), with an average age of 73.2 years, were included in the study (**Table 1**). Among these, 41 (36%) had a history of hypertension, and 37 (32.5%) had a history of smoking. Most preoperative symptoms included vision impairment (70 patients, 61.4%), followed by headache and vomiting (45 patients, 39.5%), and visual field defect (33 patients, 28.9%), (**Figure 1**). There were 84 (73.7%) non-functional pituitary adenomas (NFPAs), 10 (8.8%) lactotroph adenomas (six of whom had preoperative visual acuity loss and visual field defects, two had severe preoperative headache, dizziness, and vomiting, one had cranial nerve III palsy and one experienced consciousness disturbance), 13 (11.4%) corticotroph adenomas, and 7 (6.1%) somatotroph adenomas, (**Figure 2A**). The average tumor volume was 5.8 cm^3^, with the average longest diameter of 2.5 cm (mean ± standard deviation [SD]: 2.5 ± 0.9 cm). The study also documented seven pituitary microadenomas, seventy-one pituitary macroadenomas, and four giant pituitary adenomas. The degree of tumor aggressiveness towards the suprasellar area was quantified using the Hardy-Wilson classification. There were 42 (37.8%) patients with Grade A-C and 69 (62.2%) with Grade D-E. Likewise, the Knosp classification was used to quantify the degree of para-sellar invasion. The results showed 98 (88.3%) patients with Grade A-C and 13 (11.7%) with Grade D-E of para-sellar invasion. The degree of suprasellar invasion in the young group was significantly higher than that of parasellar invasion in these elderly patients (P<0.0001, **Figure 2B**).

**Table 1:** A summarized clinical characteristics of the 114 patients included in this study.

Clinical characteristics of the 114 patients involved				
Clinical characteristics		Score	Cases No.	Cases %
No. of patients			114	100.0
Sex	Male		65	57.0
	Female		49	43.0
Age (years)	Mean ± SD	73.2 ± 3.1		
BMI (kg/m^2^)	Mean ± SD	23.8 ± 3.2		
History of disease and comorbidities				
Incidentalomas	No		107	93.9
	Yes		7	6.1
Hypertension	No		73	64.0
	Yes		41	36.0
Diabetes mellitus	No		109	95.7
	Yes		5	4.3
Coronary heart disease	No		109	95.7
	Yes		5	4.3
Smoking	No		77	67.5
	Yes		37	32.5
Drinking	No		100	87.7
	Yes		14	12.3
Pre-op symptoms				
Vision impairment	No		44	38.6
	Yes		70	***61.4***
Visual field defect	No		81	71.1
	Yes		33	28.9
Droopy eyelids	No		109	95.6
	Yes		5	4.4
Headache & vomiting	No		69	60.5
	Yes		45	39.5
Other severe symptoms^1^	No		101	88.6
	Yes		13	11.4
VIS of vision impaired patients				***P=0.002***
Pre-op	Mean ± SD	36.4 ± 27.2		
Post- op	Mean ± SD	33.8 ± 28.5		
Type of tumor				
NFPAs			84	***73.7***
Lactotroph adenomas			10	8.8
Corticotroph adenomas			13	11.4
Somatotroph adenomas			7	6.1
Tumor volume^2^(cm^3^)	Mean ± SD	5.8 ± 5.2		
Supra- v.s. para-sella invasion degree				***P<0.0001***
Hardy-Wilson classification^3^	Grade A-C		42	37.8
	Grade D-E		69	**62.2**
Knosp classification^3^	Grade A-C		98	**88.3**
	Grade D-E		13	11.7
Surgical approach^3^				
	Endoscopic		48	43.2
	Microscopic		63	56.8
Resection degree^4^				
	NTR		90	86.5
	STR		14	13.5
Tumor apoplexy	No		103	90.4
	Yes		11	9.6
Duration of surgery (minutes)	Mean ± SD	101.2 ± 54.2		
Post-op complications				
No complications			85	74.6
Hypopituitarism	No		98	86.0
	Yes		16	14.0
Fever	No		105	92.1
	Yes		9	7.9
CSF leakage	No		110	96.5
	Yes		4	3.5
Diabetes insipidus	No		111	97.3
	Yes		3	2.7
Intracranial infection	No		113	99.1
	Yes		1	0.9
Outcome				
No recurrence			111	97.3
Recurrence			2	1.8
Dead			1	0.9
Ki-67(%)^5^	Mean ± SD	2.1 ± 2.6		
	≥3%		19	25.3
	<3%		56	74.7

^1^Including severe fatigue, anorexia, numbness of at least one limb.

^2^Relevant data of 32 patients was missing.

^3^Relevant data of 3 patients was missing.

^4^Relevant data of 10 patients was missing.

^5^Relevant data of 39 patients was missing.

Vision impairment was measured by an eye chart and visual field defect was measured by computerized perimetry. Statistically significant differences are shown in VIS of vision impaired patients, and also in supra- and para-sella invasion degree.

Bolded p-values indicate statistical significance, while bolded percentages denote the predominant categories of clinical characteristics.

Forty-eight (43.2%) patients underwent endoscopic surgery, while 63 (56.8%) underwent microscopic surgery. NTR of the tumor was achieved in 90 (86.5%) and STR in 14 (13.5%) patients. Visual improvement was observed in 20/74 patients with visual impairment and visual field defect. Preoperative VIS of these 74 patients was 36.4 ± 27.2, while post-operative VIS was 33.8 ± 28.5, and the difference was statistically significant (P=0.002, **Figure 2C**). Eleven (9.6%) patients experienced tumor apoplexy, but it did not affect preoperative and postoperative VIS (P=0.087 and P=0.151, respectively). The main post-operative complications were hypopituitarism, fever, cerebrospinal fluid (CSF) leakage, diabetes insipidus and intracranial infection. Sixteen (14.0%) patients had post-operative hypopituitarism, nine (7.9%) had a post-operative fever, four (3.5%) had post-operative nasal leakage of CSF, three (2.7%) had post-operative diabetes insipidus, and one (0.9%) had post-operative intracranial infection. The standard treatment protocol for post-operative hypopituitarism included oral hormone replacement therapy. Patients with post-operative fever were routinely managed through physical cooling and, antibiotics was administered in case of infection diagnosis. Patients with post-operative CSF nasal leakage were treated at an early stage of detection by placing the lumbar puncture and fluid drainage system which was combined with antibiotics application to prevent meningitis. Anti-diuretic treatment was given to patients who developed post-operative diabetes insipidus. One patient in the younger group required long-term oral hormone replacement therapy, while each of the above-mentioned post-operative complications was effectively managed and resolved without delay or serious consequences. Most patients (111 cases, 97.3%) had a good prognosis, however, tumor recurrence was observed in the other two patients, and one patient died due to respiratory circulatory accident during post-operative hospitalization.

### Comparison between the two groups

**Figure 3:**
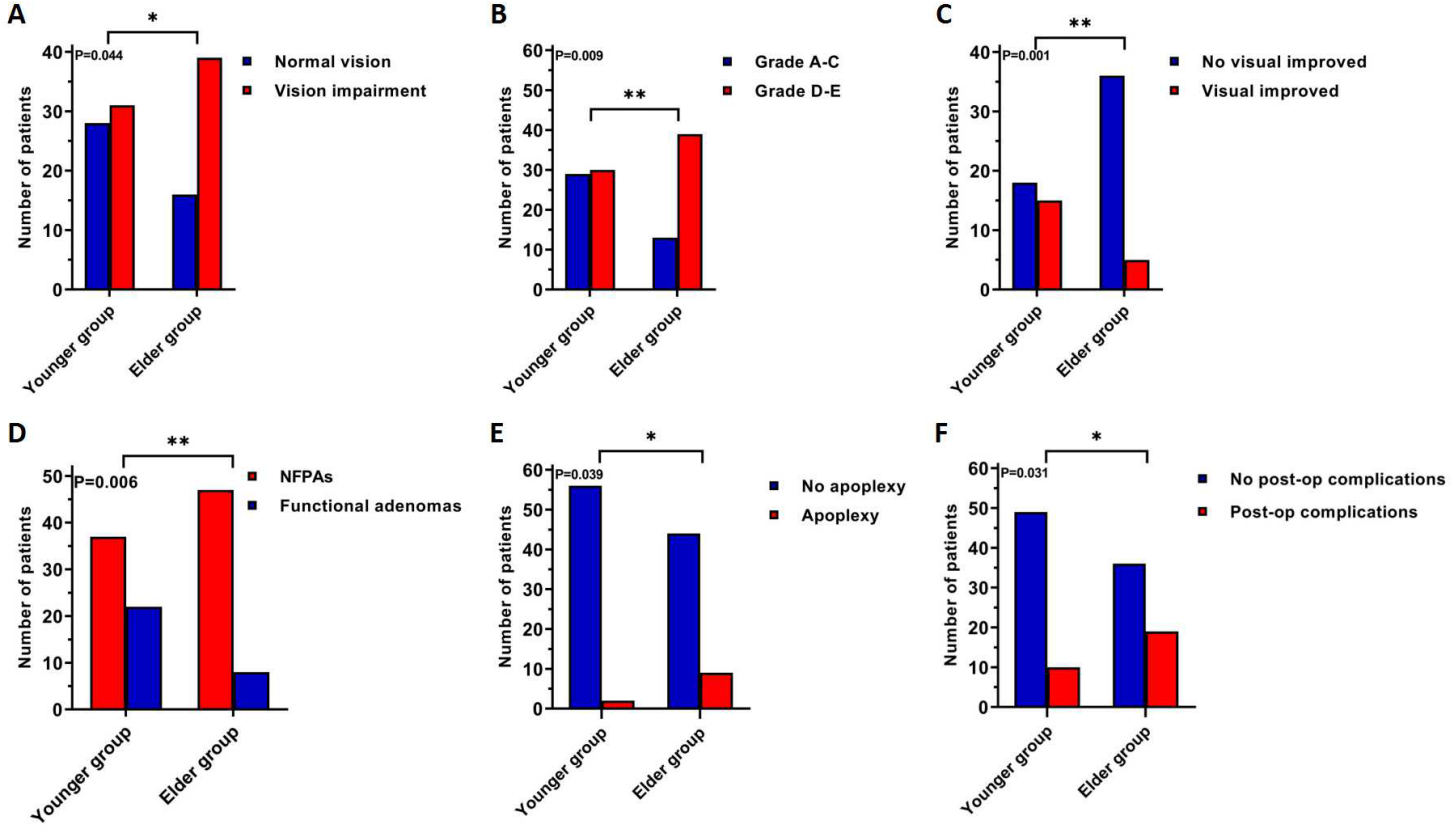
A comparison of clinical characteristics shows statistically significant differences between younger and elder groups, patients in the elderly group were more likely to have: **(A)** pre-operative visual impairment, **(B)** pre-operative supra-sella invasion **(C)** post-operative visual improvement, **(D)** NFPAs, **(E)** tumor apoplexy, **(F)** post-operative complications. *: P<0.05; **: P<0.01.

We performed a normality test on the age distribution. The skewness and peakness values were 1.324 and 1.922, respectively. Thus, based on the average age, we divided the patients were categorized into younger (age < 73 years, 59 cases) and elder (age ≥ 73 years, 55 cases) groups and statistical analysis was performed again. The results are presented in **Table 2** and **Figure 3**. Compared to the younger group, patients in the elderly group were more likely to have larger tumor volume (P=0.039), preoperative vision impairment (P=0.044, **Figure 3A**), and higher grades in the Hardy-Wilson classification (P=0.009, **Figure 3B**). Further, the post-operative visual recovery in the elderly group was effective to a lesser degree than in the younger group (P=0.001, **Figure 3C**). NFPAs in the elderly group were more common compared to the younger group (P=0.006, **Figure 3D**), as were tumor apoplexy (P=0.039, **Figure 3E**), and post-operative complications (P=0.031, **Figure 3F**). A statistical comparison revealed that no complication was more likely to occur in the elderly group (post-op hypopituitarism, P=0.218; post-op fever, P=0.421; post-op CSF leakage, P=0.110; post-op intracranial infection, P=1.000; post-op diabetes insipidus, P=1.000). However, all four patients experiencing CSF leakage were in the elderly group.

**Table 2:** A comparison between the younger and elder groups.

Comparison between the younger and elder groups					
	Younger group		Elder group		P value
	Score	Cases No.	Score	Cases No.	-
No. of patients		59		55	
Sex					0.607
Male		35		30	
Female		24		25	
Incidentalomas					0.115
No		53		54	
Yes		6		1	
Vision impairment					***0.044***
No		28		16	
Yes		31		39	
Visual field defect					0.390
No		44		37	
Yes		15		18	
Droopy eyelids					0.195
No		58		51	
Yes		1		4	
Headache&vomiting					0.785
No		35		34	
Yes		24		21	
Other severe symptoms^1^					0.296
No		50		51	
Yes		9		4	
VIS of vision impaired patients					
Pre-op	33.4 ± 24.8		38.8 ± 29.1		0.386
Post-op	29.2 ± 26.8		37.6 ± 29.5		0.207
ΔVIS (pre-op VIS - post-op VIS)	4.2 ± 5.8		1.3 ± 3.8		0.645
Post-op visual improved					***0.001***
No		18		36	
Yes		15		5	
Type of tumor					***0.006***
NFPAs		37		47	
Functional adenomas		22		8	
*Lactotroph adenomas*		*8*		*2*	
*Corticotroph adenomas*		*9*		*4*	
*Somatotroph adenomas*		*5*		*2*	
Tumor volume^2^(cm^3^)	4.5 ± 5.9		6.9 ± 4.4		***0.039***
Hardy-Wilson classification^3^					***0.009***
Grade A-C		29		13	
Grade D-E		30		39	
Knosp classification^3^					0.085
Grade A-C		55		43	
Grade D-E		4		9	
Tumor apoplexy^3^					***0.039***
No		56		44	
Yes		2		9	
Resection degree^4^					1.000
NTR		45		45	
STR		7		7	
Post-op complications		10		19	***0.031***
No complications		49		36	
Hypopituitarism					0.218
No		53		45	
Yes		6		10	
Fever					0.421
No		56		49	
Yes		3		6	
CSF leakage					0.110
No		59		51	
Yes		0		4	
Intracranial infection					1.000
No		58		55	
Yes		1		0	
Diabetes insipidus					1.000
No		57		54	
Yes		2		1	

^1^Including severe fatigue, anorexia, numbness of at least one limb.

^2^Relevant data of 32 patients was missing.

^3^Relevant data of 3 patients was missing.

^4^Relevant data of 10 patients was missing.

Vision impairment was measured by an eye chart and visual field defect was measured by computerized perimetry. The elder group was more likely to have vision problems pre- and post-operatively, a more severe suprasellar invasion, a higher proportion of NFPA, a larger tumor mass, a higher rate of apoplexy, and a worse post-operative visual improvement and comorbid postoperative complications.

Bolded p-values indicate statistical significance.

## Discussion

### Visual issues

The incidence of PA in the elderly has been reported at the rate of 4%-10% (3, 20). With the increased emphasis on life expectancy, the detection of pituitary tumors in the elderly is also increased. Nearly half of patients with NFPAs have been reported to potentially experience comorbid visual impairment, as can some hormone-secreting PAs, and a small number of patients with pituitary microadenomas (21). Elderly patients with PAs are more likely to experience symptoms of preoperative visual loss (61.4% in our study) and headache and vomiting (39.5% in our study); further, older patients were more likely to have symptoms of visual loss, which may be related to chiasmatic compression due to prolonged presence of the tumor (11–13, 22–24) and the gradual increase in symptoms was misdiagnosed as other diseases, such as cataract (3, 24–26), leading to delayed treatment (5, 9, 20). We found a correlation between the degree of preoperative visual impairment and the degree of postoperative visual improvement in elderly patients, with patients with mild preoperative visual impairment more likely to achieve postoperative improvement. The degree of visual improvement was not related to age, however, patients in the younger group were more likely to achieve visual improvement, which is consistent with the previous study (28, 29).

### Hormonal features of pituitary adenomas in the elderly

PAs in elderly patients often present as non-functional or hypopituitarism, which is usually underestimated (3, 5, 9, 11, 12, 20, 22, 25, 27, 29). NFPAs account for 1/3 of all PAs (2). The current consensus indicates that a high proportion of PAs in the elderly are NFPAs (20), consistent with the findings of the present study. According to the latest PA pathological classification, about 3/4 of the number of elderly patients had these adenomas; however, this proportion did not increase with age, possibly because the age of the study population was more than 70 years, during which the hormonal and other body functions decline, for the anterior pituitary gland undergoes fibrosis and vascular alterations (5). As a result, the proportion of functional PAs in this group was lower than in the general population. Besides, hormonal PAs are often detected in young patients because of clinical symptoms due to abnormal hormone levels; in contrast, they are usually detected in older people because of the mass effect, often caused by NFPAs (20).

### Suprasellar and parasellar invasion

**Figure 4:**
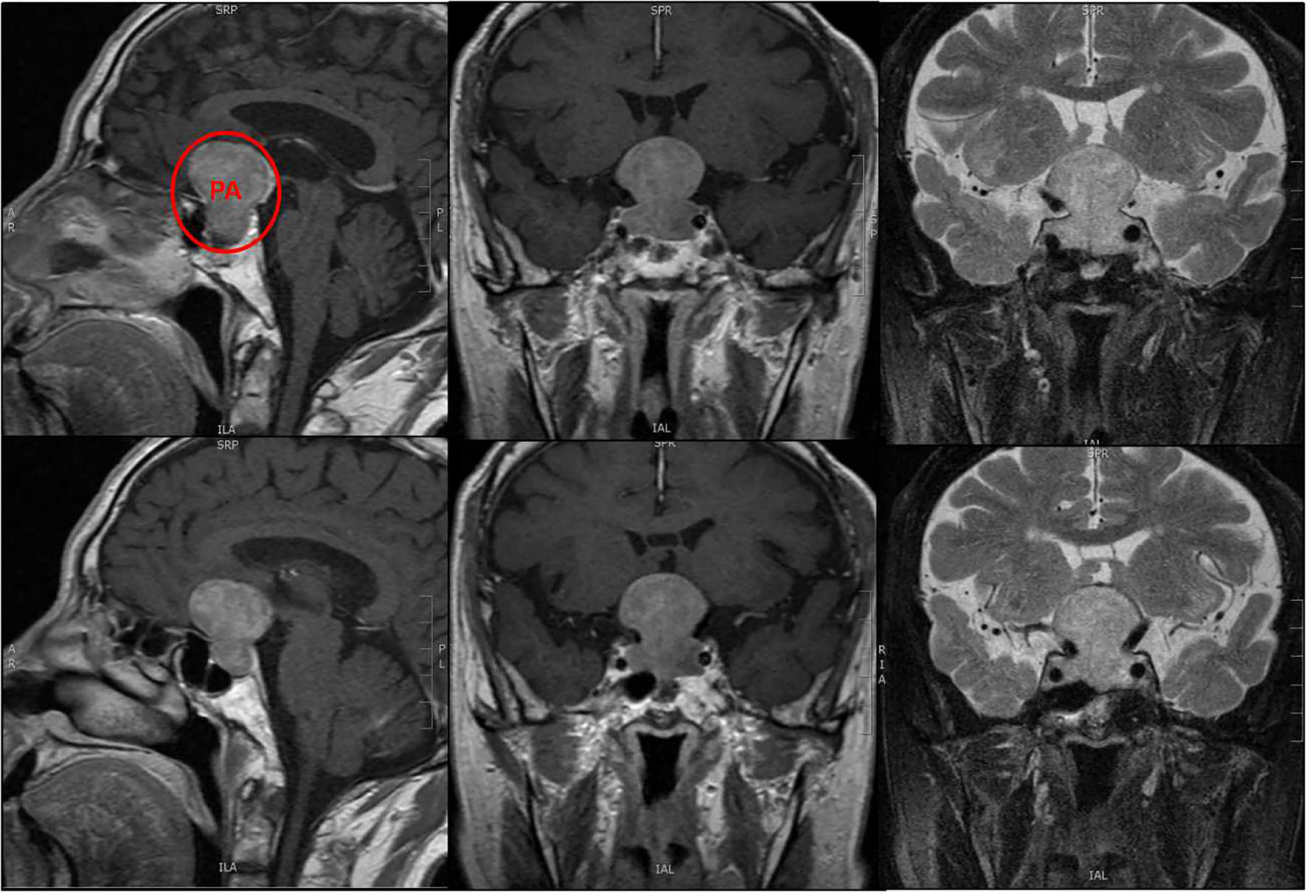
Pituitary macroadenoma in the younger group.

In the current study, tumor invasion to the suprasellar region was generally higher in elderly patients (**Figure 4**), while that to the para-sellar region was generally lower, in accordance with earlier published studies (9, 30, 31). Nonetheless, with age, tumor growth to the para-sellar region begins to occur, which may subsequently lead to palsy of the oculomotor nerves post-operatively, with prolonged post-operative recovery time (11, 12). Although vision impairment was more severe in the elderly age group, we did not find any correlation between this and the degree of suprasellar invasion of the tumor, as reported in a previous study (30). Similarly, the increase in the degree of parasellar invasion with age did not lead to any significant difference in clinical symptoms. We hypothesize that this was because the number of patients with suprasellar invasion was generally larger than those with parasellar invasion (69 cases vs. 13 cases), as a result, older patients usually consulted more for optic nerve function impairment. Increased incidence of headaches has been reported (12) in the elderly age group, considering that the mass effect of the tumor is associated with increasing age, which is in contrast to our findings and those reported in other studies (27, 32).

### Other comorbidities and complications

**Figure 5:**
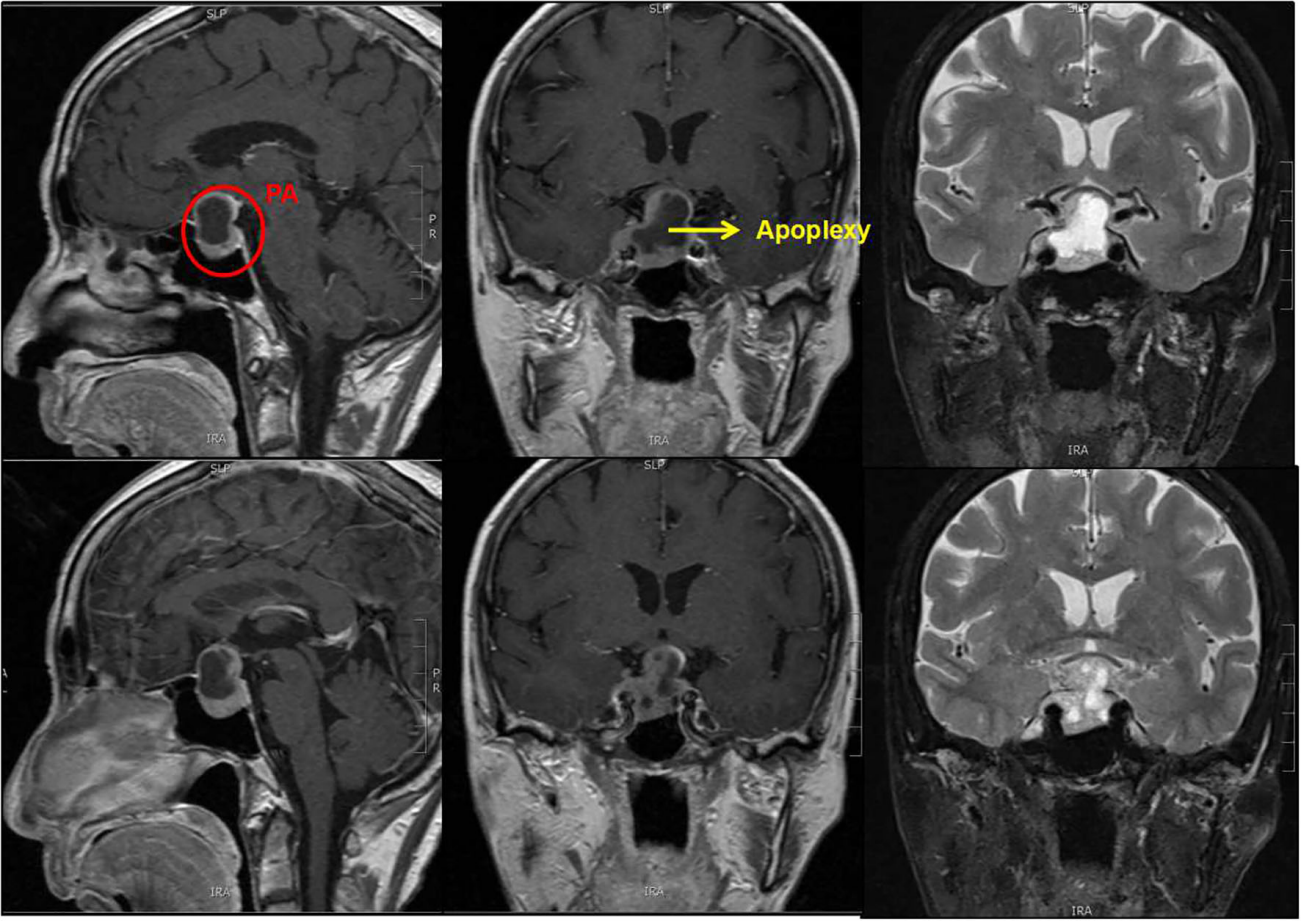
Pituitary macroadenoma in the elder group. The tumor combined apoplexy.

Consistent with the previous studies (5, 11, 23, 28, 31), there was a low probability of tumor apoplexy (9.6%, **Figure 5**), however, the risk of apoplexy increased with age (20). The mechanism of tumor apoplexy is complex, the exact factor could not be identified in this study, although previous studies have suggested that this may be associated with hypertension, coronary heart disease, or the use of anticoagulants (5, 20).

The extent of tumor resection was not affected by age (P=1.000) and the majority of patients in this study (97.3%) had no recurrence; this confirmed the effectiveness of transnasal surgery for the treatment of PAs in the elderly, as reported in other studies (3, 26, 33, 34). The probability of post-operative complications may increase with age; in fact, a relatively high level of cranial complications has been reported in elderly patients (11, 12, 31, 35), suggesting the need for aggressive early surgical treatment for symptomatic PAs in the elderly. However, this statement remains controversial (10, 13, 25, 29, 33). Transnasal endoscopic or microscopic approaches have been demonstrated to be safe and effective (7, 9, 23, 25, 30, 33), or even better in the elderly group (9, 27), while another study showed frequently increased anesthesia risk in elderly patients due to other medical conditions (3, 32). In our study, 62 (54.3%) patients had coexisting physical problems such as hypertension, diabetes mellitus, coronary heart disease, or a history of drinking and smoking that may cause pulmonary and liver function abnormalities. All patients had the ASA score in the range of I to III (39 patients were ASA I, 44 patients were ASA II, and 31 patients were ASA III, respectively). Three patients (2.7%) in our study had post-operative diabetes insipidus, all of which were transient. As there were no patients with pre-operative diabetes insipidus in earlier reports, we suggest that the presence of diabetes insipidus in this study may be related to local stimulation of the hypothalamus and/or pituitary stalk as a result of intraoperative manipulation. Post-operative diabetes insipidus is associated with injury to the hypothalamus or posterior pituitary, leading to impaired production and transport of antidiuretic hormone (33, 36). All cases of post-operative CSF leak were low-flowing, and originated from the elderly group, but the difference was not statistically significant, consistent with the previous study (7, 11, 20, 33, 34). We did not analyze the causes of CSF leaks as only four cases occurred in our study; however previous studies have shown that surgical technique is the primary causes of this complication (11, 33), in addition to the late healing of wounds in elder patients (23). Careful intra-operative manipulation to prevent the perforation of saddle septum and skull base reconstruction minimizes post-operative CSF leakage.

### Limitations

This study had a few limitations: It was a single-center retrospective study, and, given the specificity of the population, long-term follow-up could not be conducted. Therefore, subsequent multi-center prospective studies with long-term follow-up are still needed to validate the results.

## Conclusions

In this study, we found that in elderly patients with PA, visual impairment was the majority of preoperative symptoms. The tumors were frequently non-functional and associated with hypopituitarism, often showing a high degree of suprasellar invasion but minimal parasellar invasion. The correlation between post-operative visual improvement and preoperative visual acuity highlights the importance for early surgical intervention to salvage vision in elderly patients.

## Data Availability

The raw data supporting the conclusions of this article will be made available by the authors, without undue reservation.
